# Taxonomic resurrection of *Saxifraga lancangensis* (Saxifragaceae)

**DOI:** 10.1186/s40529-024-00418-y

**Published:** 2024-04-29

**Authors:** Xin-Jian Zhang, Kang-Zheng Jiang, Xin-Yuan Kuai, Jun-Tong Chen, Peng-Rui Luo, Hang Sun, Tao Deng

**Affiliations:** 1 State Key Laboratory of Plant Diversity and Specialty Crops, Kunming Institute of Botany, Chinese Academy of Sciences, Rd. Lanhei No. 132, Heilongtan, 650201 Kunming, Yunnan, China; 2 University of Chinese Academy of Sciences, 100049 Beijing, China; 3 School of Life Sciences, Yunnan University, 650500 Kunming, China

**Keywords:** Saxifragaceae, Taxonomy, Yunnan, China, Phylogeny

## Abstract

**Background:**

Accurate species delimitation is fundamental for testing evolutionary theory and provides essential implications for conservation management. The arctic-alpine genus *Saxifraga* L. (Saxifragaceae) is taxonomically complex and many species have not been critically assessed. The taxonomic and phylogenetic status of *Saxifraga lancangensis* Y.Y.Qian, considered as a synonym of *Saxifraga mengtzeana* Engl. & Irmsch. in previous studies, is re-evaluated in light of new evidence presented here.

**Results:**

Evidence from morphological comparison and sequencing of plastid genome indicate that *S. lancangensis* belongs to *Saxifraga* sect. *Irregulares* Haw., and is closely related to *Saxifraga geifolia* Balf.f., and *S. mengtzeana*. However, *S. lancangensis* can be diagnosed by its petals with red and clawless base, leaf blade orbicular and leaf margin shallowly dentate.

**Conclusions:**

The morphological and molecular evidence support the resurrection of *S. lancangensis* as a distinct species. An updated morphological description based on protologue and fresh material, diagnostic characters, and original photographs of the resurrected species are presented.

## Background

The species-rich genus *Saxifraga* L. comprises more than 400 species that are mainly distributed in arctic and mountainous regions of the Northern Hemisphere (Ebersbach et al. 2017; Pan et al. 2001; Tkach et al. 2015). *Saxifraga* is taxonomically difficult for their remarkable morphological variation, hybridization and polyploidization (Ebersbach et al. 2020; Zhang et al. 2022). Recent taxonomic revision recognized 13 sections and nine subsections within this genus (Tkach et al. 2015). *Saxifraga* sect. *Irregulares* Haw., currently encompasses ca. 22 species concentrated in eastern Asia, is one of the earliest lineages of *Saxifraga* to diverge (Tkach et al. 2015; Zhang et al. 2020b, 2021). It is well characterized by asymmetric flowers with three short and two unequally elongated petals (Magota et al. 2021; Soltis et al. 2001; Zhang et al. 2017).

In China, fifteen species of *S.* sect. *Irregulares* were recorded, including eight recently reported new species (Chen et al. 2022; Zhang et al. 2017, 2018, 2019, 2021, 2022, 2023b; Zhao et al. 2019), and a resurrected species (Zhang et al. 2023a). Most members of this section are confined to small areas (Magota et al. 2021; Pan et al. 2001), and many of them are known only from type specimens or a handful of herbarium collections. More investigations are needed to clarify the patterns of variation and the delimitation of these species.

In 2021, during an examination of *Saxifraga* specimens in the herbarium of the Kunming Institute of Botany (KUN), the first author found a specimen of *Saxifraga* identified as *Saxifraga mengtzeana* Engl. & Irmsch. (KUN 1,238,150). However, the locality of this specimen was Lancang County (Yunnan Province, China), which is out of the distribution range of *S. mengtzeana*, but coincides with the type locality of *Saxifraga lancangensis* Y.Y.Qian (Qian 1995; Zhang et al. 2023a). In 2022, Dr. Guo Yongjie shared a plant photograph of *Saxifraga* from the above locality with us. Recently, we conducted a fieldwork and collected living material of this plant from the above locality. After a comprehensive morphological comparison of the type specimens and herbarium materials and review of the relevant taxonomic literature, we confirmed that this plant represented *S. lancangensis*, which is considered as a synonym of *S. mengtzeana* in the Flora of China (Govaerts et al. 2021; Pan et al. 2001). The purpose of this study was to re-evaluate the taxonomic status of *S. lancangensis*. Based on the protologue and type specimens, *S. lancangensis* differs from *S. mengtzeana* by its petals with red base and orbicular leaf blade (Qian 1995). Geographically, the type locality of *S. lancangensis* is some 500 km distant from the type locality of *S. mengtzeana*. The flowering time of *S. lancangensis* is December to January of the next year, while the flowering time of *S. mengtzeana* is September to November (Zhang et al. 2023a). These differences suggest that the taxonomic status of *S. lancangensis* should be re-examined.

## Methods

### Morphological comparison

Morphological data were recorded based on both fresh and dried plant samples from field collections and herbarium specimens. Voucher specimens of our collections were deposited in the herbarium of the Kunming Institute of Botany (KUN), Kunming, China. Herbarium specimens of *Saxifraga* sect. *Irregulares* from the herbaria CDBI, CSFI, GXMG, HITBC, IBSC, KUN, PE, SYS, and WUK (Thiers, (updated continuously)) were examined either through direct examination of the specimens or by viewing their digital images provided by the National Plant Specimen Resource Center (https://www.cvh.ac.cn/index.php) and the JSTOR Global Plants web portal (https://plants.jstor.org/). Voucher specimens for the morphological observations of *S. lancangensis* are cited under Additional specimens examined in the Taxonomic treatment.

### Phylogenetic reconstruction

We collected 20 samples representing *S. lancangensis*, *S. mengtzeana* and related taxa (Table 1), which included 12 species of *S.* sect. *Irregulares*. *Saxifraga sinomontana* J.T.Pan & Gornall from *Saxifraga* sect. *Ciliatae* Haw. was selected as the outgroup based on previous molecular studies (Li et al. 2019; Tkach et al. 2015; Zhang et al. 2020b, 2023a). Leaf materials were collected from both field (silica-gel-dried leaves) and dry herbarium specimens. Molecular analyses were conducted using genome skimming data. The procedures for DNA extraction, library preparation, and sequencing were carried out at Novogene (Beijing, China). Sequencing libraries were generated using the NEB Next® Ultra DNA Library Prep Kit for Illumina® (NEB, USA). The prepared libraries were sequenced on an Illumina Hiseq 4000 platform with 150 bp paired-end reads. The GetOrganelle pipeline was used to assemble plastid genome data (Jin et al. 2020). Complete plastid genome was annotated in batches using the web application GeSeq (https://chlorobox.mpimp-golm.mpg.de/geseq.html) (Tillich et al. 2017). A concatenation-based approach was conducted for shared protein coding genes (PCGs) regions of the plastid genome, and sequences were aligned in MACSE v2 and trimmed by Gblocks 0.91b implemented in Phylosuite v1.2.3 (Ranwez et al. 2018; Talavera and Castresana 2007; Zhang et al. 2020a). Phylogenetic reconstruction was performed using both maximum likelihood (ML) and Bayesian inference (BI) methods. The GTR + I + G model of sequence evolution was selected as identified by jModeltest ver. 2.1.7 (Posada 2008). Maximum likelihood analysis was conducted in IQ-Tree with 1000 bootstrap (BS) replicates to estimate clade support (Bui Quang et al. 2013; Nguyen et al. 2015). Bayesian analysis was conducted in MrBayes v3.2 (Ronquist et al. 2012). Four parallel Markov Chains Monte Carlo (MCMC) simulations were run and sampled every 1000 generations for 20 million generations in total, with the first 25% trees discarded as burn-in. Runs were considered to have converged when the average standard deviation of split frequencies was less than 0.01 (Ronquist et al. 2012).

**Table 1:** Voucher information and genbank accessions for phylogenetic analysis

Taxon	Voucher	GenBank accession number
*Saxifraga lancangensis*	YNS0740 (KUN)	PP359606
*Saxifraga geifolia* 1	deng10896 (KUN)	OQ428204
*Saxifraga geifolia* 2	deng11665 (KUN)	OQ428205
*Saxifraga geifolia* 3	deng12605 (KUN)	OQ428206
*Saxifraga geifolia* 4	zwy-972 (SYS)	OQ428202
*Saxifraga mengtzeana* 1	zhangxj104 (KUN)	OQ406248
*Saxifraga mengtzeana* 2	zhangxj110 (KUN)	OQ406247
*Saxifraga mengtzeana* 3	zhangxj106 (KUN)	OQ406246
*Saxifraga mengtzeana* 4	Zhangxj177 (KUN)	OQ406249
*Saxifraga viridiflora* 1	deng12030 (KUN)	OQ428208
*Saxifraga viridiflora* 2	zhangxj98 (KUN)	NC_073566
*Saxifraga damingshanensis*	zwy-1208 (SYS)	NC_073565
*Saxifraga daqiaoensis*	deng12102 (KUN)	NC_073560
*Saxifraga shennongii*	LXP- 09- 09089 (SYS)	OQ434240
*Saxifraga kegangii*	BJ4668 (JIU)	NC_073562
*Saxifraga kwangsiensis*	deng12168 (KUN)	NC_073561
*Saxifraga luoxiaoensis*	LXP-13-16785(SYS)	NC_073563
*Saxifraga rufescens*	deng13173 (KUN)	OQ129932
*Saxifraga sinomontana*	/	MN104589
*Saxifraga stolonifera*	*/*	MN496079

## Results

### Morphology

**Fig. 1:**
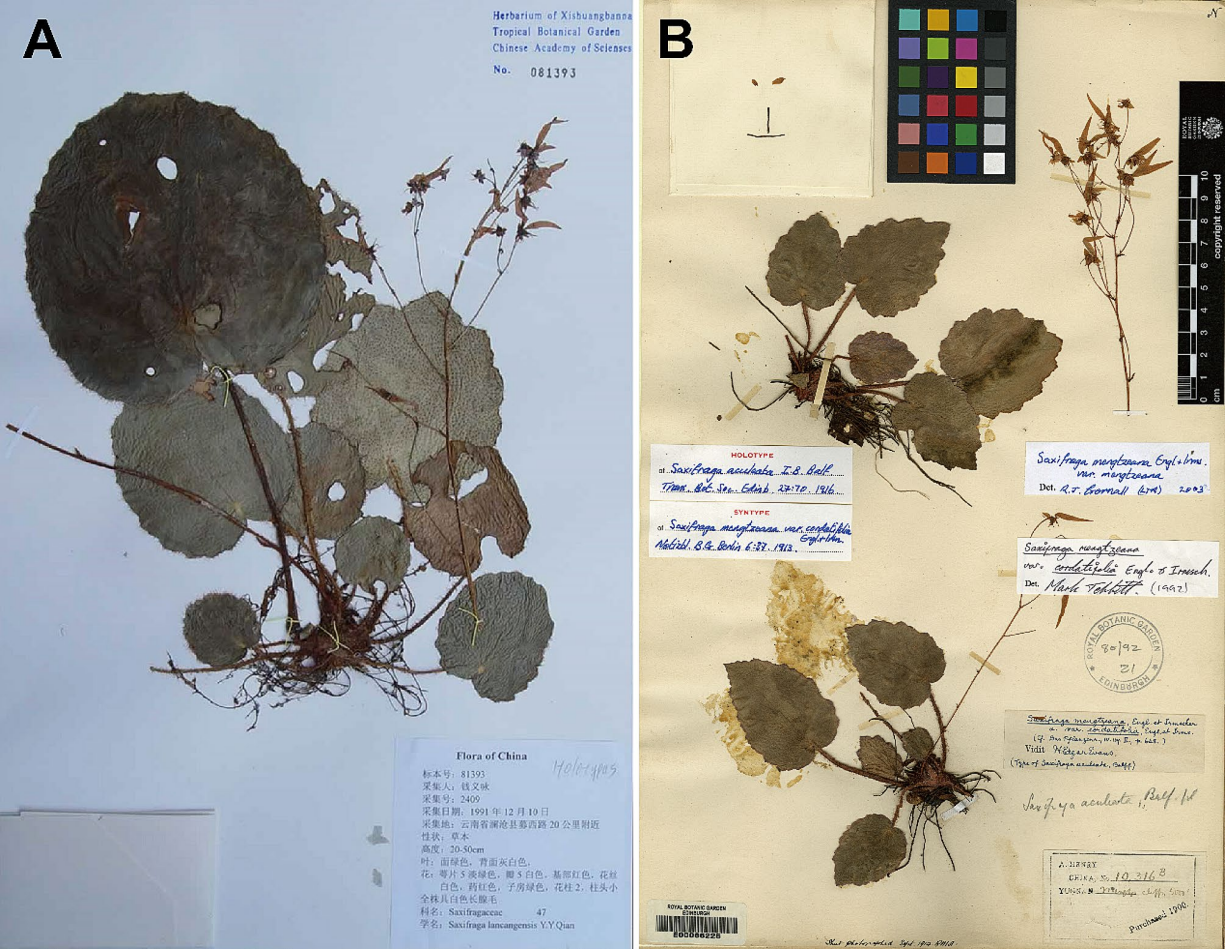
(**A**) Holotype of *Saxifraga lancangensis* Y.Y.Qian (Qian Yiyong 2409 [HITBC]) and (**B**) Lectotype of *Saxifraga mengtzeana* Engl. & Irmsch. (A. Henry 10316B [E])

**Fig. 2:**
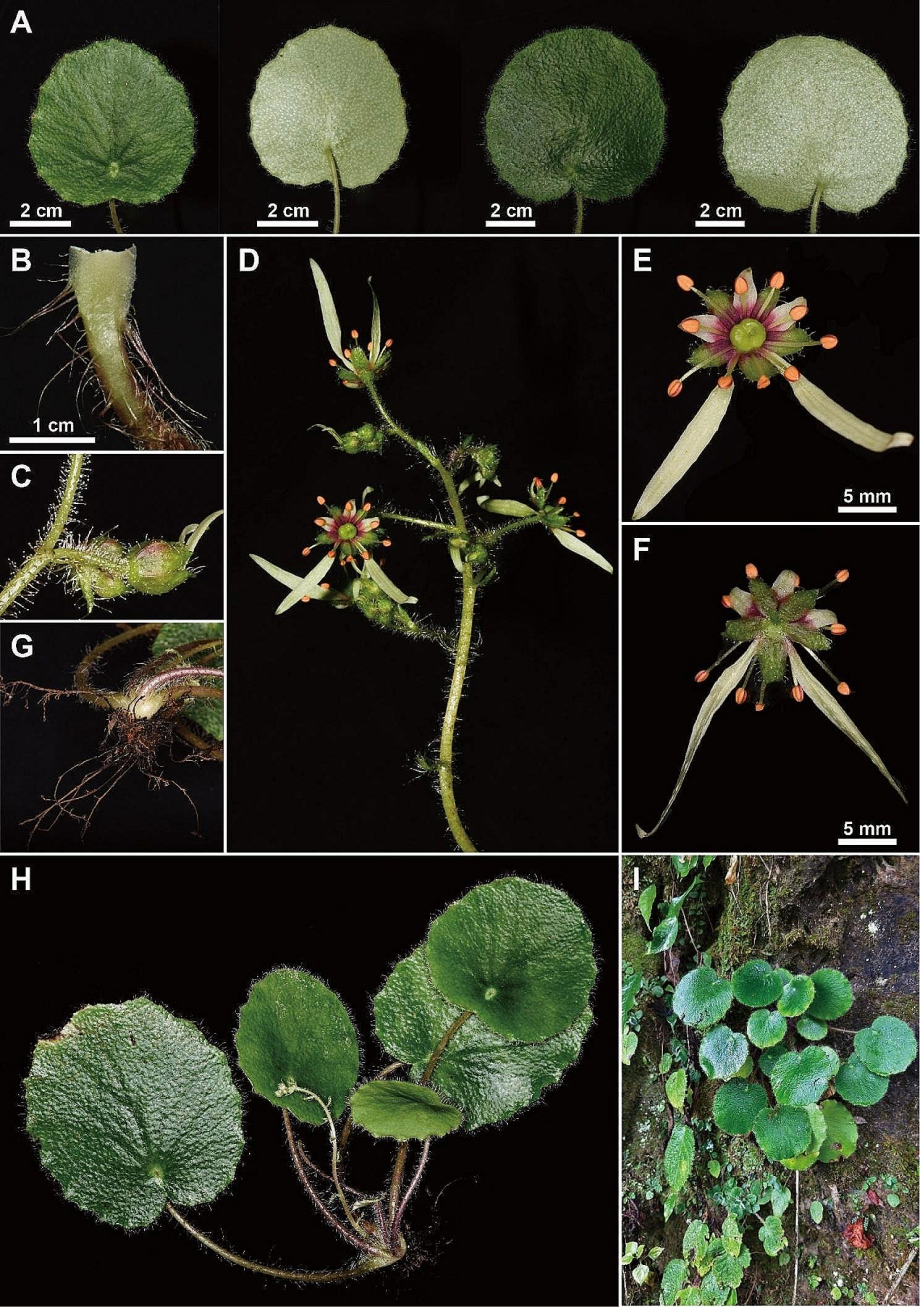
*Saxifraga lancangensis* from type locality. **A** leaf blade; **B** petiole base; **C** pedicel with white glandular pubescent; **D** inflorescence; **E** adaxial surface of flower; **F** abaxial surface of flower; **G** rhizomes; **H**&**I** plant and and habitat

**Fig. 3:**
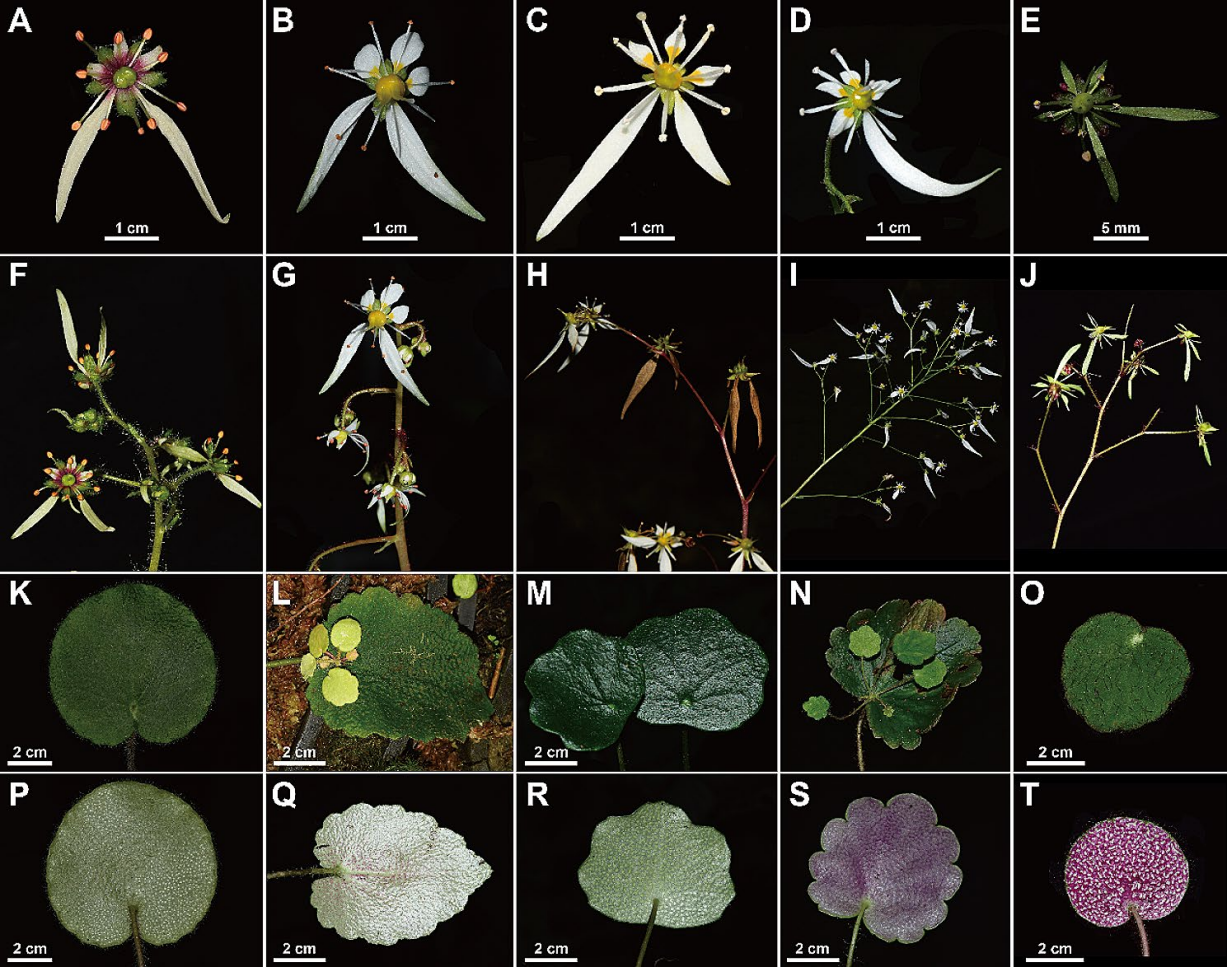
Morphological characters of *Saxifraga lancangensis* (**A**, **F**, **K**, **P**), *Saxifraga mengtzeana* (**B**, **G**, **L**, **Q**), *Saxifraga daqiaoensis* (**C**, **H**, **M**, **R**), *Saxifraga geifolia* (**D**, **I**, **N**, **S**), *Saxifraga viridiflora* (**E**, **J**, **O**, **T**). **A-E** adaxial surface of flower; **F-J** inflorescence; **K-O** adaxial surface of leaf blade; **P-T** abaxial surface of leaf blade

**Fig. 4:**
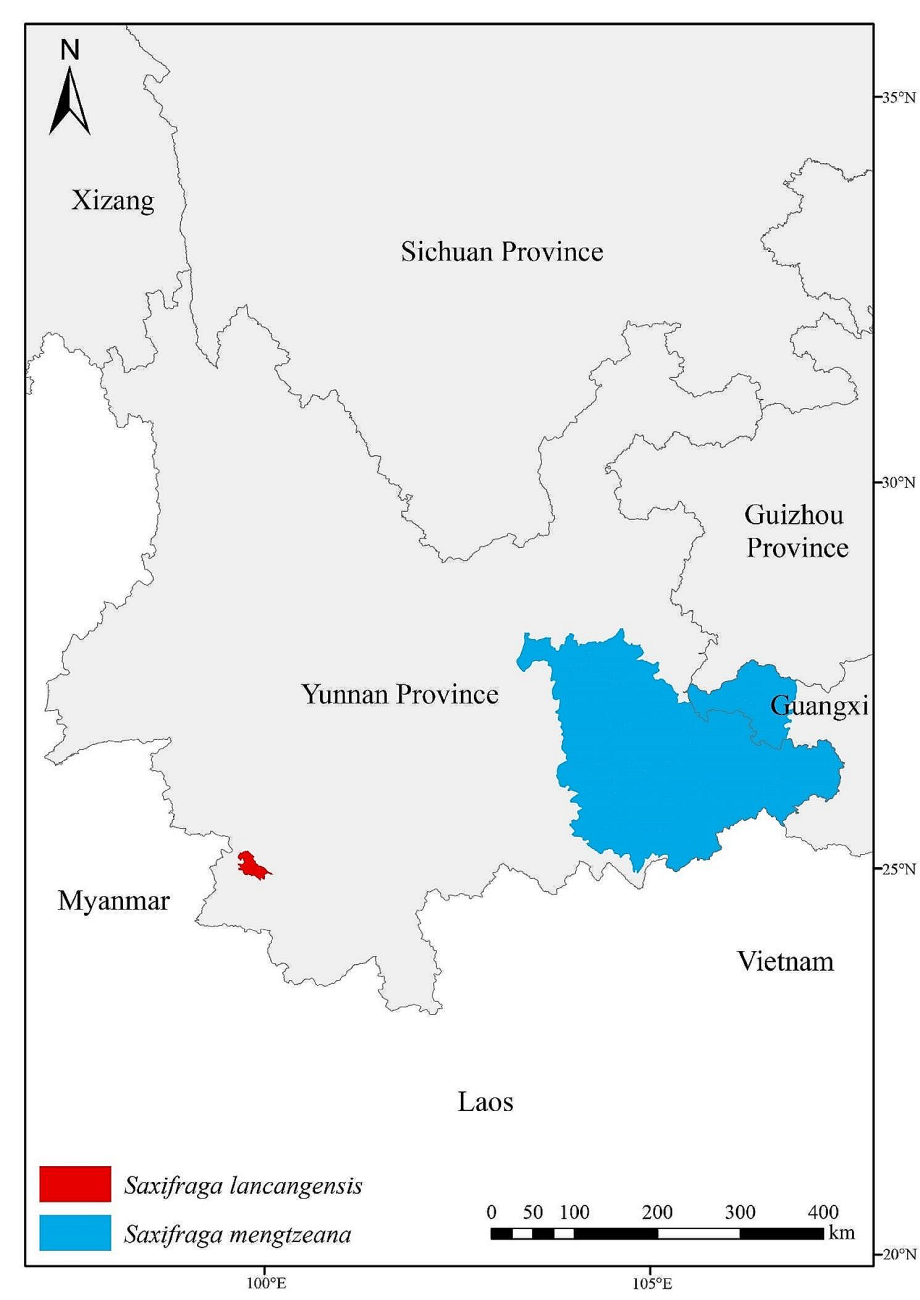
Distribution of *Saxifraga lancangensis* (red) and *Saxifraga mengtzeana* (blue) in southwestern China. Prepared by Xin-Yuan Kuai

The asymmetric flowers and absence of stolons (Figs. 1 and 2) of *Saxifraga lancangensis* indicate a position in *Saxifraga* sect. *Irregulares* ser. *Rufescentes* J.T. Pan (Pan 1991; Pan et al. 2001). Morphological comparison of *S. lancangensis* and known species of *S.* sect. *Irregulares* indicates that *S. lancangensis* resembles *S. mengtzeana* and *Saxifraga daqiaoensis* F.G.Wang & F.W.Xing in having leaf blade with a peltate petiole insertion (Figs. 2 and 3), which distinguish them from other species in *S.* sect. *Irregulares*. It is notable that the peltate feature is plastic in *S. lancangensis*, and there are intermediates with the basifixed phenotypes within the same population, which is consistent with our observations on *S. mengtzeana* and *S. daqiaoensis* (Zhang et al. 2023a). Besides, Balfour (1916) described *Saxifraga henryi* Balf.f. on account of its peltate leaves, but it was now accepted as part of the variation encompassed by *S. mengtzeana* (Pan et al. 2001; Zhang et al. 2023a). *S. lancangensis* is distinct from the latter two chiefly in its petals with a red and clawless base, and its orbicular leaf blade. The morphological comparisons of these species are presented in Table 2. Geographically, the distribution ranges of these three species are isolated from each other (the type locality of *S. lancangensis* is about 500 km distant from the type locality of *S. mengtzeana*, and some 1300 km distant from the type locality of *S. daqiaoensis*) (Fig. 4).

**Table 2:** Diagnostic characters of *Saxifraga lancangensis* and comparison with other related species of *Saxifraga* sect. *Irregulares*

Characters	*S. lancangensis*	*S. daqiaoensis*	*S. mengtzeana*	*S. geifolia*	*S. viridiflora*
Leaf shape	orbicular	reniform-cordate	triangular-cordate	reniform	reniform
Leaf margin	shallowly dentate	shallowly crenately-lobed to subentire	crenate-dentate	crenate-lobed	undulate to subentire
Trichomes on leaf	both surfaces sparsely hispid or nearly glabrous	adaxially sparsely hispid, abaxially glabrous	both surfaces sparsely hispid or nearly glabrous	both surfaces glandular hispid or nearly glabrous	both surfaces crisped villous
Spots on abaxial leaf surface	brown spots	brown spots	brown or greenish spots	brown spots	white spots
Trichomes on petiole	glandular hairy	glabrous	brown glandular hairy	brown glandular hairy	crisped villous
Trichomes on inflorescence	branches glandular pubescent	branches nearly glabrous	branches glandular pubescent	branches glandular pubescent	branches glandular pubescent
Petal	white to greenish, base red, base of small petals clawless	white, yellow spotted at base, base of small petals contracted into a claw	white, yellow spotted at base, base of small petals contracted into a claw	white, yellow spotted at base, base of small petals contracted into a claw	green, base unspotted, base of small petals contracted into a claw
Nectary disc	greenish	yellow	yellow	yellow	obscure
Flowering time	December to January	March to May	September to November	May to September	April to July

### Phylogenetic analyses

**Fig. 5:**
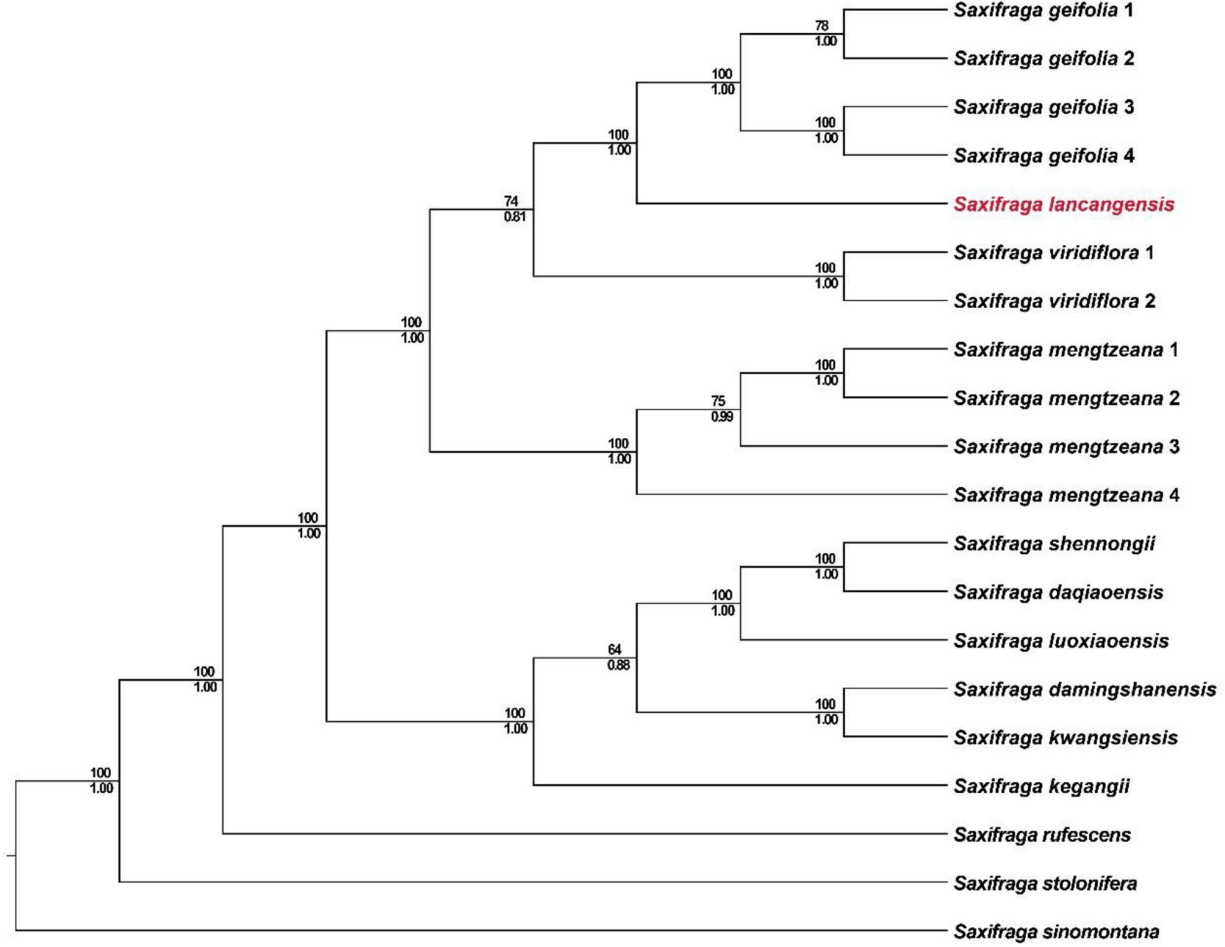
The phylogenetic position of *Saxifraga lancangensis* in *Saxifraga* sect. *Irregulares* derived from the combined 73 shared protein coding genes (PCGs) regions of the plastid genome, with *Saxifraga sinomontana* as outgroup. Numbers above branches indicate ML bootstraps, numbers below branches are Bayesian posterior probability

A total of 13 taxa were included in the phylogenetic analysis using 73 shared protein coding genes (PCGs) regions of the plastid genome of 20 samples. The resulting concatenated matrix dataset contained 60,472 bp. The 50% majority-rule consensus tree (Fig. 5) based on maximum likehood bootstraps (ML) and Bayesian posterior probability (PP) of PCGs both revealed a sister relationship between *S. lancangensis* and *Saxifraga geifolia* Balf.f. with strong supports (ML = 100, PP = 1). The monophyletic clade of *S. lancangensis* and *S. geifolia* grouped together with *Saxifraga viridiflora* X.J.Zhang, T.Deng, J.T.Chen & H.Sun, and were sister to *S. mengtzeana* (ML = 100, PP = 1).

## Discussion

*Saxifraga lancangensis* was listed among the synonyms of *S. mengtzeana* in the Flora of China, which are widely accepted in reference lists such as POWO (https://powo.science.kew.org/) and Tropicos (https://tropicos.org/home). In the current study, the taxonomic status of *S. lancangensis* was re-evaluated by conducting morphological comparison and phylogenetic analysis of this species and related species. Morphological (Table 2) and molecular (Fig. 5) evidence are consistent in distinguishing *S. lancangensis* from *S. mengtzeana*. Overall, *S. lancangensis* is easily distinguished from any other species of *Saxifraga* sect. *Irregulares* by its petals with a red, clawless base. Additionally, *S. lancangensis* differs markedly from others species in *S.* sect. *Irregulares* not only by morphological characteristics and geographical distance, but also by flowering time. Most species of *S.* sect. *Irregulares* bloom in summer or autumn, while *S. lancangensis* flowers in winter. The differentiation in flowering time may play an important role in the reproductive isolation of the species in *S.* sect. *Irregulares*.

### Taxonomic treatment

#### Saxifraga lancangensis

Y.Y.Qian, Acta Bot. Austro Sin. 10: 70–72 (1995) (Figs. 1 and 2).

#### Type

CHINA. Yunnan Province, Lancang County, Zhutang Town, alt. 2000 m, 10 Dec. 1991, *Qian Yiyong*, *2409* (Holotype HITBC!, isotype IBSC! ).

#### Description

Perennial herbs, 10–35 cm tall. Rhizomes short. Stolons absent. Leaves all basal, forming a rosette; petiole 5–15 cm long, fleshy, glandular hairy, base sheathed; leaf blade orbicular, leathery and slightly succulent, 6.5–11.5 cm long × 5.5–9.5 cm wide, both surfaces sparsely hispid or nearly glabrous, abaxially greenish, with brown spots, base cordate or peltate, margin shallowly dentate, apex obtuse or acute. Inflorescence paniculate, 15–25 cm long. 10–30-flowered; branches 2.0–6.0 cm long, glandular pubescent, 2–5-flowered; pedicels slender, 1.0–2.0 cm long, glandular pubescent. Flowers asymmetric; sepals 5, spreading, oblong-ovate, 4.0–5.0 mm long × 1.5–2.0 mm wide, adaxially glabrous, abaxially and marginally glandular pubescent. Petals 5, white to greenish, base red, margin entire; shortest 3 petals ovate, 4.0–4.5 mm long × 1.7–2.0 mm wide, base truncate, apex acute; two long petals narrowly ovate to lanceolate, 10.0–22.0 mm long × 2.0–3.5 mm wide. Stamens 10, 5.0–7.0 mm long. Ovary ovoid, 1.5–2.0 mm long, with a semiannular greenish nectary disc; styles divergent ca. 2.0–4.0 mm long.

#### Phenology

Flowering was observed from December to January of the next year, and fruiting from January to March.

#### Distribution and habitat

*Saxifraga lancangensis* is native to Zhutang Town, the eastern parts of Lancang County, Yunnan Province. It grows in limestone at altitudes 1780–2000 m under evergreen broad-leaved forest dominated by *Neocinnamomum delavayi* (Lecomte) H.Liu, *Cornus oblonga* Wall., *Piper sarmentosum* Roxb. and *Elatostema salvinioides* W.T.Wang.

#### Comparison with close *Saxifraga* species

Peltate-leaved feature was found in *S. lancangensis*. There are only two known species with peltate petiole insertions in *S.* sect. *Irregulares*—*S. daqiaoensis* and *S. mengtzeana*. *S. lancangensis* differs from latter two species in flower color (petals white to greenish, base red vs. petals white, base with yellow spots), petals shape (base of small petals clawless vs. base of small petals contracted into a claw), nectary disc (greenish vs. yellow). *S. lancangensis* is also distinct from *S. mengtzeana* in leaf shape (orbicular vs. triangular-ovate), leaf margin (shallowly dentate vs. crenate-dentate), flowering time (December to January of the next year vs. September to November). *S. lancangensis* is also distinguished from *S. daqiaoensis* by leaf shape (orbicular vs. reniform-cordate), trichomes on petiole (glandular hairy vs. glabrous), flowering time (December to January of the next year vs. March to May). Molecular phylogenetic analyses indicated a close relationship within *S. lancangensis*, *S. geifolia* and *S. viridiflora*. However, *S. lancangensis* can easily be distinguished from latter two species by its petals with red base. Additionally, *S. lancangensis* is also different from *S. geifolia* in leaf margin (shallowly dentate vs. crenate-lobed), flowering time (December to January of the next year vs. May to September). *S. lancangensis* is also distinguished from *S. viridiflora* by petal color (white to greenish vs. green), sepals (green, glandular pubescent, without verruculose vs. red, glabrous, abaxially white verruculose), trichomes on leaf (nearly glabrous vs. crisped villous), flowering time (December to January of the next year vs. April to July). Geographically, *S. viridiflora* is an endemic species of Guilin, Guangxi Province, and *S. geifolia* is restricted to northern Yunnan and Sichuan, both are more than 500 km distant from the type locality of *S. lancangensis*. It is worth noting that *Saxifraga cataphracta* X.J.Zhang, T.Deng & H.Sun is also closely related to *S. geifolia* (Zhang et al. 2023b), however, *S. cataphracta* morphologically differs from other species of *S.* sect. *Irregulares* by its abaxial leaf surface covered with white or virescent streaks. The capsule beaks of *S. cataphracta* is winged when mature (vs. capsule beaks divergent in *S. geifolia* and *S. lancangensis*). *S. cataphracta* is known from northeastern Yunnan, Chongqing and southern Sichuan, which is more than 500 km distant from the geographical distribution of *S. lancangensis*.

#### Additional specimens examined: CHINA

Yunnan Province, Lancang County, 22 Feb. 1992, *Qian Yiyong*, *2493* (Paratype HITBC!, IBSC! ); Yunnan Province, Lancang County, limestone under forest, 24 Oct. 1989, *Tao Guoda, Li Xiwen, 39,839* (HITBC); Yunnan Province, Lancang County, Zhutang Town, alt. 2015 m, 28 Jan. 2010, *Hu Qihe, Zhao Qiang, Zhou Ying, Zhang Shaoyun*, *YNS0740* (KUN); Yunnan Province, Puer City, 19 Mar. 2012, *Zhang Shaoyun, Ye Jinke, Hu Qihe, YNS1206* (KUN); Yunnan Province, Lancang County, Zhutang Country, alt. 1833 m, 8 Dec. 2023, *Deng Tao, Zhang Xinjian, lc-7* (KUN).

#### Conservation significance

*Saxifraga lancangensis* is an endemic species with narrow geographical distribution only known from Zhutang Town, Lancang County of Yunnan, China. It grows in limestone under forest close by the road, which can be easily disturbed. We estimate that the population size at the type locality comprises less than 300 individuals. Due to its limited range, fragile habitat and low population size, *S. lancangensis* should be considered as a protected plant in China.

## Data Availability

The sequences of this study have been deposited in The National Center for Biotechnology Information (NCBI) database. GenBank accession numbers of the sequencing data can be found in Table 1.

## Abbreviations

BI: Bayesian inference
BS: Boostrap
CDBI: Chengdu institute of biology, chinese academy of Sciences, Herbarium
CSFI: Central-south forestry university, herbarium
GXMG: Guangxi medicinal botanic garden, herbarium
HITBC: Xishuangbanna tropical botanical garden, academia sinica, herbarium
IBSC: South china botanical garden, chinese academy of sciences, herbarium
KUN: Kunming institute of botany, chinese academy of sciences, herbarium
MCMC: Markov chains monte carlo
ML: Maximum likelihood
PE: Institute of botany, chinese academy of sciences, herbarium
PCG: Protein-coding gene
PP: Posterior probability
STEP: Second tibetan plateau scientific expedition and research program
SYS: Sun Yat-sen University, Herbarium
WUK: Northwestern Institute of Botany,, Chinese Academy of Sciences, Herbarium
